# Hippocampal Malrotation: A Genetic Developmental Anomaly Related to Epilepsy?

**DOI:** 10.3390/brainsci11040463

**Published:** 2021-04-05

**Authors:** Ting-Ying Fu, Chen-Rui Ho, Chih-Hsiang Lin, Yan-Ting Lu, Wei-Che Lin, Meng-Han Tsai

**Affiliations:** 1 Department of Pathology, Yuan’s General Hospital, 162 Cheng Hung 1st Road, Kaohsiung 80249, Taiwan; f6588888@yahoo.com.tw; 2 Department of Neurology, Kaohsiung Chang Gung Memorial Hospital, 123 Dapi Road, Niaosung District, Kaohsiung 83301, Taiwan; ultima1229@cgmh.org.tw (C.-R.H.); thsign@adm.cgmh.org.tw (C.-H.L.); yantimlu@yahoo.com.tw (Y.-T.L.); 3 Department of Radiology, Kaohsiung Chang Gung Memorial Hospital, Kaohsiung 83301, Taiwan; alex@cgmh.org.tw; 4 School of Medicine, Chang Gung University, 259 Wenhau 1st Road, Taoyuan 33302, Taiwan

**Keywords:** Hippocampal malrotation, epilepsy, MRI, Hippocampal Sclerosis, genetics

## Abstract

Hippocampal malrotation (HIMAL) is an increasingly recognized neuroimaging feature but the clinical correlation and significance in epilepsies remain under debate. It is characterized by rounded hippocampal shape, deep collateral, or occipitotemporal sulcus, and medial localization of the hippocampus. In this review, we describe the embryonic development of the hippocampus and HIMAL, the qualitative and quantitative diagnosis issues, and the pathological findings of HIMAL. HIMAL can be bilateral or unilateral and more on the left side. Furthermore, the relevance of HIMAL diagnosis in clinical practice, including its role in epileptogenesis and the impact on the pre-surgical decision are reviewed. Finally, the relationship between HIMAL and hippocampal sclerosis (HS) and the possible role of genetics in the etiology of HIMAL are discussed. The evidence so far suggested that HIMAL does not have a significant role in epileptogenesis or surgical decision. HIMAL could be a genetic developmental imaging feature that represents a more diffuse but subtle structural error during brain development. Many questions remain to be explored, such as possible cognitive alteration associated with HIMAL and whether HIMAL predisposes to the development of HS. Further studies using high-quality MRI, unified consensus qualitative and quantitative diagnostic criteria, and comprehensive cognitive assessment are recommended.

## 1. Introduction

Hippocampal malrotation (HIMAL), also termed incomplete inversion of the hippocampus or hippocampal “malformation,” is an increasingly recognized neuroimaging finding of undetermined clinical significance. HIMAL was initially identified in patients with malformations of cortical development (MCD) and/or agenesis of the corpus callosum [1,2]. Subsequently, isolated HIMAL was also found in patients without an obvious brain malformation [3]. Since the hippocampus is one of the most important brain structures associated with epilepsy, HIMAL has been implicated in the development of epilepsy; however, the relationship is not clear cut.

## 2. Terminology

The term “hippocampal malformation” is somewhat confusing in the pathological and imaging literature because it has been used in different ways, including a broader concept of any abnormality during hippocampal development, which included HIMAL and hippocampal hypoplasia. Some authors have used “hippocampal malformation” or “hippocampal developmental abnormalities” to describe conditions similar to HIMAL [4,5]. Others have used the term “malformation” to denote a small or hypoplastic hippocampus with a normal T2 signal [6,7]. The smaller hippocampus may be associated with a blurred internal structure and/or a flattened hippocampal head or body, which may progress to become hippocampal sclerosis (HS) [7]. However, in HIMAL, the hippocampus usually has a normal T2 signal but appears to be rounded or pyramidal in shape. It is unclear whether these different findings are just different variants of the same abnormal development of the hippocampus or represent completely different etiologic entities. In this review, the term HIMAL refers to those cases fulfilling specific MRI features instead of the ambiguous term “hippocampal malformation.”

## 3. Normal Development of Human Hippocampus

The insight of how HIMAL develops relies on the knowledge of normal hippocampal development from histopathology and MRI studies [8,9,10]. During the gestational age 13–14 weeks, the hippocampus is located along the medial surface of the temporal lobe with an unfolded appearance and surrounded by a widely open hippocampal sulcus. At 15–16 weeks, the dentate gyrus and cornu ammonis start to “roll” into the medial temporal lobe, but the hippocampal sulcus remains open. The parahippocampal gyrus, especially the subicular region, is larger and more medially positioned. The CA1, CA2, and CA3 areas of the cornu ammonis are arranged linearly. The dentate gyrus has a narrow U shape. At age 18–21 weeks, the dentate gyrus and cornu ammonis embed into the temporal lobe as the hippocampus and subiculum approximate each other across a narrow hippocampal sulcus. The CA1–3 fields form an arc and the CA4 field increases in size within the widened arch of a C-shaped dentate gyrus forming an interlocking structure. At the end of this stage, the fetal hippocampus begins to resemble the adult hippocampus [9]. However, this inversion process is usually not symmetric. A recent fetal MRI study demonstrated that in most cases the right hippocampus develops faster [11]. Postnatal MRI studies show that the volume of the hippocampus increases rapidly until the age of two years and continues to increase slowly thereafter till adolescence [12]. Some studies reported that the right hippocampus is larger than the left [12,13,14]. After adolescence, there is a trend of a gradual decrease in hippocampal size with age [13].

## 4. Mechanisms of HIMAL Development

The resemblance of HIMAL to the fetal hippocampus leads to the hypothesis that HIMAL results from the failure of the normal infolding process during development. The two possible mechanisms for incomplete infolding are as follows:

- Lack of infolding drive: The hippocampal infolding process is passively driven by the development of the neocortex, which pushes the hippocampus deeper into the temporal lobe forming the “Swiss roll” appearance. If there was a problem with neocortical development, the hippocampus would assume its prenatal position. This is likely the explanation for HIMAL associated with diffuse cortical malformations, for instance, periventricular heterotopia, polymicrogyria, and lissencephaly;
- Local blockage or tectonic effect: The maldevelopment or disorganization of the CA1/Subiculum forms a “blockade” or “tectonic plate,” which impedes or disrupts the infolding process of the hippocampus [4,15]. This would explain why the neocortex appears normal in isolated HIMAL.

The exact cause of the failure is unknown. Acquired factors, such as toxins, metabolism derangement, or ischemia, in combination with genetic factors occurring during this critical stage of brain development, are likely contributing to the pathogenesis of HIMAL.

## 5. MRI Features and Qualitative Diagnosis of HIMAL

**Figure 1:**
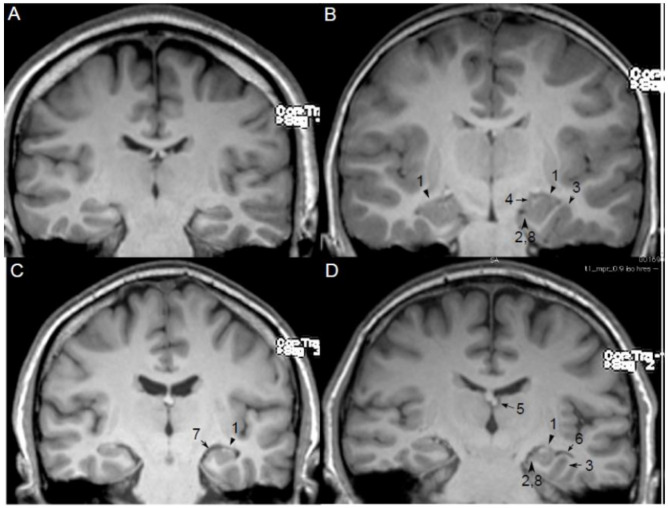
MRI qualitative features of hippocampal malrotation (HIMAL). Figure 1 Legend: (**A**) Normal bilateral hippocampus, (**B**) Bilateral HIMAL, (**C**,**D**) Unilateral HIMAL on left side. 1 Round or pyramidal shape instead of ovoid shape, 2 medial position of hippocampus on hippocampal sulcus, 3 excessively deep or “verticalized” collateral sulcus, 4 medial located fimbria, 5 small or displaced fornix, 6 enlarged temporal horn, and empty choroid fissure, 7 thickened subiculum, 8 reduced upper horizontal portions of the parahippocampal gyrus.

Several MRI findings have been described as features of HIMAL (Figure 1) [3,16,17,18,19], which include the following:

1. Round or pyramidal shape instead of ovoid shape;
2. Medial position of the hippocampus on the hippocampal sulcus;
3. The collateral sulcus is excessively deep or “verticalized”;
4. Fimbria located medial to the hippocampus;
5. Small or displaced fornix;
6. Enlarged temporal horn and empty choroid fissure;
7. Thickened subiculum;
8. Reduced upper horizontal portion of the parahippocampal gyrus.

However, there is no consensus regarding the diagnostic criteria for HIMAL. Not all proposed features are present in every case, and only a few cases have all the features described. Most previous studies did not specify clearly how HIMAL was defined [3,16,17,19]. This is probably because of the lack of a “gold standard” for HIMAL diagnosis such as its pathology. Many studies used the consensus of two experienced neuroradiologists as the definite diagnosis of HIMAL; however, this is also variable among different groups [5,20]. This problem is highlighted in a recent meta-analysis of HIMAL in which moderate heterogeneity was found across reviewed studies [21]. 

Barsi et al. initially described 10 characteristics associated with HIMAL; some features are not specific and can also be observed in healthy individuals (e.g., normal temporal lobe size, normal corpus callosum, and abnormal position and size of fornix) [22]. Unilateral involvement was excluded because bilateral HIMAL can be observed in some patients in subsequent studies [5,20,23,24]. Later, Bernasconi et al. used eight criteria (described above) to evaluate the hippocampal formation in patients with MCD and temporal lobe epilepsy (TLE). They found that all eight criteria were more frequently seen in patients with MCD patients and seven criteria (except rounded and vertical hippocampus) were more commonly seen in patients with TLE, compared to normal controls [18]. At least three criteria were found in 49% patients of MCD and 43% patients of TLE, compared to 10% of healthy controls. This study did not investigate the sensitivity or specificity of each criterion in terms of the diagnosis of HIMAL. 

Using the radiologists’ consensus diagnosis, Tsai et al. studied the brain MRI scans of 103 healthy volunteers and found that 1. rounded shape, 2. verticalization of the dominant inferior temporal sulcus (DITS), and 3. flattened lateral margin of the hippocampus were the three qualitative features that were significantly associated with HIMAL [20]. The reason for using DITS instead of the collateral sulcus (CS) is because we noted that CS is not always the deepest sulcus in the inferior temporal lobe; sometimes, occipitotemporal sulcus (OTS) is the predominant sulcus. Despite reaching statistical significance, the verticalization of DITS is also commonly seen in patients with the normal hippocampus, hence not specific for HIMAL. Other qualitative features were not significant for HIMAL and can be seen in the normal hippocampus. Similarly, Labate et al. also explored this issue with Bernasconi’s eight criteria and found that hippocampal shape, medial hippocampal position, and reduction of the upper portion of the parahippocampal gyrus are sensitive and specific for the diagnosis of HIMAL [5]. Verticalization of CS had good specificity but was less sensitive. In conclusion, the best imaging feature to diagnose HIMAL appears to be the shape of the hippocampus among all the studies. The diagnosis of HIMAL heavily depends on human visual analysis of qualitative traits, which is useful in clinical practice but also subjective to bias. Therefore, several groups developed quantitative analysis for HIMAL to avoid potential observer bias. 

## 6. Measurements of HIMAL

**Figure 2:**
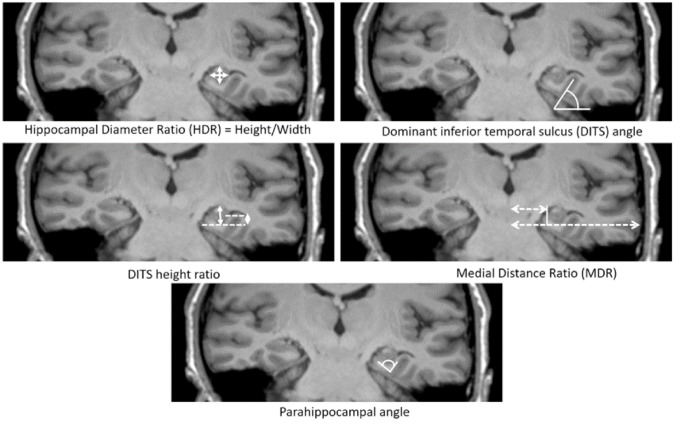
Quantitative assessments of medial positioning and vertical orientation of hippocampus. Figure 2 Legend: Hippocampal diameter ratio (HDR) was calculated by measuring the height and width of hippocampal. This represents the shape change of HIMAL; the normal hippocampus usually has a smaller ratio than HIMAL. Dominant inferior temporal sulcus (DITS) refers to the predominant sulcus of the inferior temporal surface, either collateral sulcus (CS) or occipitotemporal sulcus (OTS). This is because there are variations among individuals; some have very shallow CS, and the predominant sulcus is OTS instead. DITS angle was the angle of the sulcus from the horizontal line, which represents the verticalization of DITS. DITS height ratio was the height from the inferior margin of the hippocampus to the superior margin of the DITS divided by the total hippocampal height. The DITS height ratio represents the deep sulcus that protrudes to an empty temporal horn. The medial distance ratio (MDR) was calculated by measuring the distance between the midline and the fimbria divided by the total distance from the midline to the temporal neocortex passing through the temporal horn of the lateral ventricle. MDR represents the medial localization of the hippocampus. Parahippocampal angle (PHA) was calculated by measuring the angle between the descending and ascending portion of the parahippocampal gyrus. PHA also represents the shape change of the hippocampus (Figure adapted from [18,20]).

In the past decade, several quantitative methods for HIMAL have been developed (Figure 2). Bernasconi et al. used medial distance ratio (MDR) and parahippocampal angle (PHA) to represent the medial localization and verticalization of the hippocampus [18]. Tsai et al. expanded the quantitative methods using five additional quantitative measurements—hippocampal diameter ratio (HDR), DITS angle, DITS height ratio, MDR, and the PHA [20]. In a blinded analysis of 103 healthy volunteers, only three measures (HDR, DITS height ratio, and PHA) were significantly associated with the diagnosis of HIMAL. These features correspond to a more rounded hippocampus, deep DITS and hippocampal verticalization observed in qualitative traits. The quantitative assessment of HIMAL is more objective than visual criteria and is recommended for future studies on this subject, for example, a recent study of HIMAL in juvenile myoclonic epilepsy (JME) [23]. More studies are needed to determine the utility of using quantitative measurements to facilitate the diagnosis of HIMAL.

## 7. Histopathology of HIMAL

The histopathology of HIMAL, which is different from well-documented HS, has not been extensively studied. This is probably because HIMAL is not well recognized nor a surgical target. Baulac et al. first described a case of left HIMAL on MRI with a predominant left temporal focus on EEG who underwent surgery. The histopathology showed an opened hippocampal fissure and destruction of the normal cytoarchitecture of the subicular area of the parahippocampal gyrus in addition to features of HS including the neuronal loss in the hilus, CA1, and CA3 area, and dispersion of granular cells of the dentate gyrus [3]. Thom et al. reported a postmortem case of a 47-year-old man with bilateral (left more prominent than right) HIMAL on MRI before sudden unexplained death [4]. In contrast to Baulac’s report, no features of HS were found. The main finding was the complex folding of the pyramidal cell layer of the CA1 region involving the hippocampal body, which had an excessively long and serpentine appearance. The same group later reported two autopsy cases with long-standing seizures and the same pathologic findings [6]. Similarly, Sloviter et al. identified a histopathologic pattern termed a “tectonic” hippocampal malformation in 16 surgical patients with TLE [15]. These hippocampi contained bulbous expansions of CA1 pyramidal/subicular layers that invaginated, at times bisecting entirely, the adjacent dentate gyrus. Neuronal loss was less extensive than in HS. Dericioglu et al. also reported a single case with bilateral HIMAL and right TLE, who became seizure free on medication after right temporal lobectomy. The pathology revealed atypical convolution of the CA1/subicular region without obvious cell loss and an angulated shape of the dentate gyrus caused by indentation of CA1 [25]. Overall, the pathological findings of HIMAL appear to be distinct from HS and mainly involve the organization of hippocampal structure, particularly in the CA1 region.

## 8. Laterality of HIMAL

HIMAL can be bilateral or unilateral, and it appears to be more often bilateral in MCD patients. It has been suggested that when the cortical malformation (polymicrogyria, heterotopia, or schizencephaly) is unilateral, the HIMAL tends to occur on the same side [26]. However, a recent study of 76 MCD patients found no correlation between the two [18]. In terms of laterality, HIMAL is more likely to have occurred on the left side (~65%) in all the reported studies (Table 1). It is uncertain why a preference for this side occurs. One possible explanation is that if the development of the right hippocampus starts earlier and is completed more rapidly, intercurrent factors associated with failed infolding could have a more detrimental effect on the left hippocampus because of the later development [19].

## 9. Epileptogenic Role of HIMAL 

A relationship between HIMAL and epilepsy has been implicated, especially whether it is an epileptogenic lesion. Yeghiazaryan et al. reported successful surgical treatment of two cases with left HIMAL and left TLE. Matsufuji et al. also reported five epilepsy patients with unilateral HIMAL, and they suggested that HIMAL could have a causative role in these cases [27,28]. However, later studies found that HIMAL can also be observed in the normal population [18,19,20,29]. This brings up the possibility that HIMAL occurred by chance on the same side in these cases and was not causally related to their seizures [18,19]. As summarized in Table 2, which details the previously reported series of HIMAL and epilepsy, the lack of a definitive association between the side of HIMAL and seizure onset argues against HIMAL being epileptogenic. However, these studies may be limited by publication bias, inconclusive seizure localization, and heterogeneous subjects including individuals with extra-temporal epilepsies.

On the other hand, HIMAL was found more commonly in various types of epilepsy patients (6–43%) than in normal controls (0–18%) in some studies, although this was not limited to particular epilepsy syndromes, including focal epilepsy, intractable epilepsy, MCD, and JME [2,19,22,24,30]. Bernasconi et al. used at least three of eight predefined criteria and found that HIMAL was more common in both MCD (49%) and TLE (43%) groups than in controls (10%). Labate et al. studied 187 patients with medial TLE and 93 healthy controls using similar criteria (>3 of the eight criteria), HIMAL was found more common in the TLE group than in controls (18.8% versus 8.6%) (Labate et al., 2020). HIMAL has also been reported in up to a third of patients with MCD, particularly periventricular heterotopia and polymicrogyria [21]. Moreover, the HIMAL tended to occur on the same side of the MCD if unilateral [26]. Sato et al. retrospectively studied patients with congenital brain malformations and showed that patients with epilepsy had a higher rate (74%, 14/19) of HIMAL than brain malformation patients without epilepsy (46%, 13/28) [26]. This suggests that HIMAL is related to brain malformation and increases seizure susceptibility. A recent study on JME, the most common generalized epilepsy syndrome, also identified more HIMAL in JME patients and unaffected siblings than in controls [23]. This led to the speculation that HIMAL might be a heritable imaging marker associated with an increase in overall seizure susceptibility.

In contrast, Tsai et al. studied 155 MRI negative TLE and 103 healthy controls, in which HIMAL was more commonly observed in the epilepsy group than in controls (24.3% versus 16.1%) but not statistically significant. A recent meta-analysis of 591 patients from seven studies also reported only a weak increase in odds (1.69) of HIMAL in epilepsy patients [21]. The fact that HIMAL can also be observed in up to 18% of healthy individuals argues against a strong role in epileptogenesis.

Moreover, studies using cognitive function tests, morphological, and functional MRI provided further evidence of a more diffuse extrahippocampal developmental process in association with isolated HIMAL [31,32]. For example, Cury et al. found that patients with HIMAL displayed extra-hippocampal morphological changes in sulci mainly located in the limbic lobe [33]. Caciagli et al. showed that JME patients with HIMAL exhibited different activation pattern for visual and verbal memory, which suggest that HIMAL may exert subtle functional alternation on temporal or extratemporal cognitive networks. [23]. Thus, several authors surmised that HIMAL could be a marker of a more extensive developmental brain disorder and may contribute to seizure susceptibility [18,26]. Overall, the evidence thus far suggests that HIMAL is not a strong epileptogenic lesion for various epilepsy syndromes. Whether HIMAL represents a subtle regional or diffuse cortical developmental problem and acts as a weak risk factor for epilepsy remains to be explored. The significance of finding HIMAL in the diagnosis of epilepsy remains uncertain. Whether novel imaging technology can differentiate epileptogenic HIMAL from non-epileptogenic HIMAL is a question to be answered.

## 10. The Role of HIMAL on Pre-Surgical Decision

Temporal lobe epilepsy (TLE) is a more common focal epilepsy in adults. These patients are good surgical candidates if not responding to antiseizure medications (ASMs). The possibility of HIMAL being epileptogenic raised an important clinical question as to whether HIMAL should be considered as an imaging marker (such as HS) to inform epilepsy surgery. We performed a study on 155 lesion-negative TLE patients and identified 25 patients with HIMAL (11 bilateral, 12 left, and 2 right). There was no correlation between the side of seizure onset and the side of HIMAL [20]. This suggests that the presence of HIMAL in medication refractory TLE patients should not influence the decision of epilepsy surgery. Leech et al. also studied a smaller cohort (*n* = 48) of surgically treated pediatric patients and found that HIMAL did not predict the surgical side and outcome [34]. In conclusion, there is insufficient evidence to use HIMAL for guiding epilepsy surgery in both adult and childhood TLE or other epilepsy syndromes.

## 11. Relationship between HIMAL and HS

Another unresolved matter is the relationship between HIMAL and HS. Fernadez et al. reported two families with febrile seizures (FS) in which the probands had temporal lobe epilepsy and HS. Some affected individuals with FS and asymptomatic relatives had hippocampal malformation [7]. However, the “malformation” in this paper refers to asymmetric small hippocampi rather than the abnormal shape and orientation that typify HIMAL. Depondt et al. also reported a family with temporal lobe epilepsy and febrile seizures linked to chromosome 12q22-23.3, in which some affected and unaffected individuals had HIMAL [35]. Bernasconi et al. also found no association between the side of HIMAL and the side of hippocampal atrophy [18]. However, it is arguable whether analyzing the shape and position of the hippocampus is reliable when hippocampal atrophy exists. It has been hypothesized that HIMAL may increase susceptibility to febrile seizures, leading to the formation of HS and subsequent epilepsy. On the contrary, Sen et al. reported two patients with MCD and long-standing epilepsy who had HIMAL on MRI but did not develop HS on pathological examination. They concluded that HIMAL may not necessarily develop into HS, although they also could not exclude this could happen in other patients [6]. Recently, the large prospective Consequences of Prolonged Febrile Seizures in Childhood (FEBSTAT) study found that HIMAL is more commonly seen in patients with febrile status epilepticus than in patients with simple febrile seizures [36]. They concluded that HIMAL indicates an abnormality during brain development that predisposes to febrile seizures. Although febrile status epilepticus is a well-known risk factor for the development of HS, no robust evidence shows that HIMAL evolves into HS in humans.

## 12. Genetics of HIMAL

How genes are involved in the development of the hippocampus or HIMAL is still obscure. Some families with HIMAL or case reports associated with genetic variations have been reported. Families with predominantly febrile seizures may include some family members with HIMAL [7,35]. Kobayashi et al. described a family with two brothers presenting with 15q trisomy, both with minor dysmorphic features, mental retardation, epilepsy, and bilateral HIMAL on MRI [37]. Pramparo et al. reported a patient with a 22q13 duplication who presented with bipolar disorder, dysmorphic features, and unilateral HIMAL, but other intracranial MRI abnormalities were also described, including high signal in the periventricular white matter, hippocampal atrophy (on the other side), and hypoplastic corpus callosum [38]. Andrade et al. reported a high prevalence (64%) of unilateral HIMAL in 19 consecutive cases with 22q11.2 microdeletion. However, the presence of HIMAL in patients both with and without epilepsy argues against its contribution to epileptogenesis in this cohort [39]. Sisodiya et al. reported four patients with de novo heterozygous *SOX2* mutations who presented with anophthalmia and bilateral HIMAL; two had seizures [40]. *SOX2* is a good candidate gene for HIMAL since it is vital to the developing brain and eye. However, screening of patients with a variety of different epilepsy syndromes failed to show any variants. Additionally, no genes have been associated with “isolated” HIMAL thus far. The recent JME study has provided further insight into the genetics of HIMAL [2]. Both JME patients and unaffected siblings were significantly more likely to have HIMAL than controls, suggesting that HIMAL is a heritable imaging trait that contributes to the polygenic composition of JME. 

From animal models, several genes have been associated with the development of the mouse hippocampus including *Wnt3a*, *Emx2*, and *Lhx5* genes [41,42,43]. The genetic factors involved in the process of embryonic hippocampal development are largely still unclear. The interpretation of these results concerning human hippocampal development awaits further correlation. Conversely, it is also possible that HIMAL is determined by environmental factors during fetal brain development without a major genetic component. A better understanding of the genetic factors involved during human hippocampal development will help clarify this matter.

## 13. Debates about Normal and Abnormal Hippocampus

One interesting issue regarding HIMAL is whether it is a “normal” variant or an “abnormal” structural lesion. The dichotomy is obviously arbitrary and not pragmatic in terms of understanding the pathophysiology. Just as some “normal” individuals may have epileptiform discharges on their EEG and never have seizures, they represent the continuum of different degrees of seizure susceptibility below the clinical threshold. The hippocampal structural variation is likely to form a spectrum spanning from the typical mature “ovoid” shape to the immature “fetal” configuration and those in between. There is still uncertainty about how to define an “abnormal” hippocampal structure based on current knowledge. This requires more studies of “normal” subjects and careful correlation with the clinical phenotype (not only seizures but also other cognitive functions known to be associated with the hippocampus such as memory or broader neuropsychiatric traits).

## 14. Conclusions

There are still many questions that remain to be explored about HIMAL in terms of diagnosis, its relationship with epilepsy, and its pathogenesis. Studies have demonstrated that HIMAL is not a good presurgical imaging marker for epilepsy surgery. However, HIMAL still could be a structural variant that represents an underlying brain developmental problem that predisposes to epilepsy to some degree and may have a genetic underpinning. Tackling these questions requires studies with high-quality MRI imaging, unified diagnostic criteria, and comprehensive cognitive evaluation.

## Figures and Tables

**Table 1:** Laterality of HIMAL in reported series.

Studies	Cohort	*n*	left	Right	Bilateral
Baulac et al., 1998 [3]	Epilepsy	19	9	5	5
Barsi et al., 2000 [17]	Epilepsy	32	22	6	4
Thom et al., 2002 [4]	Epilepsy	1	0	0	1
Sen et al., 2005 [6]	Epilepsy	2	0	1	1
Bajic et al., 2007 [19]	Controls	19	13	0	6
Bajic et al., 2009 [24]	Epilepsy	60	40	4	16
Bajic et al., 2009 [24]	Controls	28	20	0	8
Dericioglu et al., 2009 [25]	Epilepsy	1	0	0	1
Yeghiazaryan et al., 2010 [27]	Epilepsy	2	2	0	0
Matsufuji et al., 2012 [28]	Epilepsy	5	2	3	0
Tsai et al., 2016 [20]	Lesion-negative TLE (*n* = 155) and Healthy Controls (*n* = 103)	50	26	8	21
Caciagli et al., 2019 [23]	JME (*n* = 37), sibling (*n* = 16) and controls (*n* = 20)	30	22	3	5
Labate, Sammarra et al., 2020 [5]	MTLE (*n* = 187) and controls (*n* = 93)	38	30	6	2
Total		287	186 (64.8%)	36 (12.5%)	70 (24.4%)

**Table 2:** Relationship between sides of seizure onset and HIMAL.

Studies	Subjects	Seizure Onset Side	Findings of HIMAL	Comments
Bernasconi et al., 2005 [18]	13/30 TLE patients	The clinical decision from multiple types of investigations	85% bilateral or contralateral versus 15% ipsilateral (*p* = 0.01)	HIMAL was not related to the side of the EEG focus
Bajic et al., 2009 [24]	14/57 TLE patients (11 left, 2 right, and 1 bilateral)	Interictal EEG only	1. 8 of 11 patients with left HIMAL had EEG focus on the left and 2 with bilateral EEG focus. 2. 2 of 2 patient with right HIMAL had left EEG focus 3. 2 patients (1 bilateral HIMAL, 1 left HIMAL) had undetermined EEG side	This study concluded that the laterality of EEG onset did not correlate with HIMAL.
Barsi et al., 2000 [17]	32 epilepsy patients with HIMAL	Interictal EEG only	8/32 (25%) contralateral 11/32 (34%) multifocal or bilateral 13/32 (45%) ipsilateral	Same as above
Thom et al., 2002 [4]	1 TLE patient with left HIMAL	Clinical semiology suggested left-sided onset (ictal right head deviation)	Ipsilateral (*n* = 1)	Supports that HIMAL is epileptogenic but potential publication bias
Sen et al., 2005 [6]	2 epilepsy patients (1 right, 1 bilateral HIMAL)	Left-sided onset based on Todd’s paralysis and interictal EEG, 1 non-localizing (bilateral, R > L HIMAL)	Ipsilateral in one patient (*n* = 1)	Supports that HIMAL is epileptogenic but potential publication bias
Yeghiazaryan et al., 2010 [27]	2 TLE patients with left HIMAL	VEM and left ATL, both became seizure-free	2 ipsilateral	Supports that HIMAL is epileptogenic but potential publication bias
Matsufuji et al., 2012 [28]	5 HIMAL (2L, 3R) cases (3 BECTS, 1 FLE, 1 undetermined)	Interictal EEG only	4 Ipsilateral, 1 uncertain (Diffuse spike and waves)	Supports that HIMAL is epileptogenic but potential publication bias
Tsai et al., 2016 [20]	25 TLE patients with HIMAL from 155 lesion-negative TLE patients	Clinical semiology, electroencephalography, and VEM	9 ipsilateral all on left side, 5 contralateral (2 right HIMAL and left-sided seizure onset, 3 left HIMAL and right-sided seizure onset), 11 bilateral HIMAL (9 left-sided seizure onset and 2 right-sided seizure onset)	Exact binomial test not significant, which suggests that the occurrence of HIMAL and seizure onset side did not differ from chance. HIMAL is not ictogenesis in lesion negative TLE and should not influence the surgical decision.
Caciagli et al., 2019 [23]	37 JME, 16 unaffected siblings, 20 controls	Generalized	22 left, 3 right, and 5 bilateral	HIMAL is not likely to be epileptogenic in JME.
Labate et al., 2020 [5]	187 MTLE patients and 93 controls	Interictal EEG only	11/19 patient with left HIMAL has left-sided EEG focus 3/4 patients with right HIMAL, EEG focus were on the left side 3/6 bilateral HIMAL, EEG focus on the left side	Concordance EEG focus with the side of HIMAL was presented in slightly more than half (52.2%) of the MTLE patients
